# Who Seeks Treatment When Medicine Opens the Door to Pathological Gambling Patients—Psychiatric Comorbidity and Heavy Predominance of Online Gambling

**DOI:** 10.3389/fpsyt.2017.00255

**Published:** 2017-11-29

**Authors:** Anders Håkansson, Emma Mårdhed, Mats Zaar

**Affiliations:** ^1^Psychiatry, Department of Clinical Sciences Lund, Faculty of Medicine, Lund University, Lund, Sweden; ^2^Malmö Addiction Center, Clinical Research Unit/Gambling Disorder Unit, Skane Region, Malmö, Sweden

**Keywords:** gambling, gambling disorder, pathological gambling, comorbidity, gender, online gambling

## Abstract

**Background:**

Few studies have assessed treatment-seeking behavior and patient characteristics in pathological gambling focusing on psychiatric comorbidity, particularly in a setting of heavy exposure to online gambling. This study aimed to address patient characteristics in a novel health care-based treatment modality for pathological gambling, including potential associations between gambling types, psychiatric comorbidity, and gender.

**Methods:**

All patients undergoing structured assessment between January 2016 and April 2017 were included (*N* = 106), and patient records were reviewed for cooccurring psychiatric disorders and types of problem games.

**Results:**

Eighty percent were men, and 58% received a psychiatric disorder apart from pathological gambling. Problematic gambling on online casino and online sports betting represented 84% of patients. Non-substance-related psychiatric comorbidity was significantly associated with female gender.

**Conclusion:**

Online gambling is more clearly predominating in this setting than in studies from other countries. High rates of comorbidity call for structured psychiatric assessment in problem gambling, with a particular focus on female patients with pathological gambling.

## Introduction

Pathological gambling is a condition affecting roughly 0.5% of the adult population (1, 2), and up to 3–5% of adults are reported to suffer from past-year problem gambling with or without fulfilment of diagnostic criteria (3). Despite documented knowledge about effective treatment for the disorder (4), several potential barriers may limit treatment-seeking behavior in gambling patients. For example, it has been suggested that in individuals with pathological gambling, treatment-seeking may—paradoxically—be inversely associated with gambling severity (5). In addition to individual factors complicating treatment-seeking, structural and organizational issues also may constitute barriers to treatment for individuals with problem gambling (6), and clearly, widespread availability is crucial to treatment coverage.

Sweden, in contrast to extensive epidemiological studies of gambling in the population (7, 8), is an example of a setting where no national strategy for gambling treatment hitherto has been established. National policies for allocation of gambling treatment have been lacking, and few regions have established actual structured treatment for problem gambling, such that in many settings throughout the country, no actual treatment has been available (9). At the same time, Sweden is seeing a vast increase in media advertisements of primarily online gambling and online sports betting, and an increased market share of overseas online gambling operators. This trend has called for action in gambling prevention and treatment by policy makers (10).

Online gambling could potentially be associated with a higher risk of problem gambling, expressed as symptom criteria of pathological gambling, compared to more traditional, land-based gambling (11–14). However, this is not a consistent finding (13), and other data indicate that online gamblers may be less likely to be categorized as problem gamblers (15). Also, it has been suggested that individuals engaging in problem gambling online may be less likely to seek treatment than problems users of land-based gambling modalities (13), and the proportion of online gamblers among treatment-seeking gamblers has been reported to be low (16). In recent years, there is an increasing attention to psychiatric comorbidities in gambling patients with or without treatment (16), and data are mixed as to whether rates of psychiatric comorbidity in online gamblers are higher or lower (7, 13, 16, 17). Gainsbury argued that poor health and substance use may be more common in online gamblers (13). On the other hand, Hing and coworkers reported lower rates of psychological distress among online gamblers than among land-based gamblers (16). Blaszczynski and coworkers reported that exclusive online gamblers were less likely to be problem gamblers, and less likely to suffer from psychological distress compared to land-based gambles (17). Also, in a Swedish population survey conducted in 2008, psychological distress was less common in Internet gamblers (7), whereas Jiménez-Murcia and coworkers identified no difference in psychopathology between land-based and online gamblers (18).

This study is based on the first 16 months with a regional, out-patient facility established within the public health care system, and in a setting where such treatment for pathological gambling previously has been unavailable in the medical sector (9). The aim of this study was to assess the characteristics of treatment-seeking patients in a treatment modality new to this setting. Particularly, given the vast exposure to online gambling advertising in the media in this setting (10), we aimed to study the extent of online gambling as opposed to more traditional gambling in treatment-seeking patients in a new treatment setting and to investigate whether these different gambling modalities differed with respect to gender and prevalence of psychiatric comorbidity.

## Materials and Methods

### Participants

Included subjects in this study are people seeking treatment voluntarily for problem gambling from January 2016 until April 2017, and if they either were assessed by the physician (the first author of this paper, Anders Håkansson) or underwent an intake assessment by one of the nurses (Emma Mårdhed and Mats Zaar).

### Materials

The study is based on hospital records from the treatment episode of the gambling facility at Malmö Addiction Center, as well as from treatment contacts occurring in parallel or in relevant temporal proximity to this episode. All relevant and recent hospital records for the patients were thoroughly reviewed by the first author.

The following data were available in patient records; age (rounded off to entire years), gender, way of accessing the facility (self-referral, referral from primary care, or referral from psychiatry), problematic types of gambling reported and the main type of gambling, and psychiatric disorders fulfilled. For the diagnosing of the gambling problem, diagnostic criteria from the DSM-5 were applied, based on the criteria often applied in research and described in documentation from the Swedish Public Health Agency (10, 19), as judged by the physician (Anders Håkansson). These criteria include the fulfillment of four or more of the following criteria: need to gamble with increasing amounts of money, being restless or irritable when trying to cut down, repeated unsuccessful attempts to stop gambling, preoccupation with gambling, gambling when feeling distressed, “chasing losses,” lying about gambling, jeopardizing, or losing relationships or work, etc., and the relying on others for money. In this study, all subjects met these criteria. Although based on DSM criteria, given the Swedish health care setting where diagnoses are made and registered as ICD-10 codes, the disorder was coded as the pathological gambling diagnosis (F63.0) according to this diagnostic system (international classification of diagnoses, 10th edition, WHO) diagnosis pathological gambling (F63.0). In the description of psychiatric comorbidity, diagnoses were included in the study if they either were diagnosed by the physician (Anders Håkansson), or from another physician at a parallel treatment unit, or by a physician at a recent health care contact which was judged by the first author to have relevance to the current treatment episode. Thus, historical diagnoses were not registered.

As the association between gender and psychiatric comorbidities in problem gamblers may differ for sub-groups of disorders; mood disorders may be more common in female gamblers, and substance use disorders more common in male gamblers (20). For this reason, psychiatric comorbidity was assessed both for the entire group of disorders, and for non-substance-related disorders separately. For both comparisons, the comorbidity variable was reported as the presence or absence of any psychiatric disorder (or any non-substance-related psychiatric disorder).

For gambling types, these were *a priori* categorized as online casino gambling, online sports betting, Internet poker, land-based casino gambling, land-based electronic gambling machines (EGMs), land-based sports and horse racing, bingo and others (including face-to-face card games and stock market).

### Design and Procedure

This study is a retrospective patient chart review from an out-patient facility for the treatment of pathological gambling in the public specialized health care system in the Skane region in Sweden. Skane is a region with approximately 1.3 million inhabitants, and this facility is located in Malmö, the capital city of the region with approximately 300,000 inhabitants. The gambling facility is one of the sub-units of Malmö Addiction Center, responsible for out-patient, emergency, and in-patient treatment of addictive disorders related to alcohol, illicit drugs, pharmaceuticals, and gambling. This unit is an out-patient facility, providing voluntary treatment. This facility opened in January, 2016, in a setting where gambling-related problems previously were not treated or assessed in the health care system, but where treatment was provided by the social authorities in the city of Malmö. In addition, free-of-charge peer-to-peer support was offered by the national patient organization, by its local facility in Malmö.

This out-patient facility at Malmö Addiction Center provides medical assessment and treatment based on cognitive-behavioral therapy (CBT) and relapse prevention (RP). Psychological treatment is provided by two nurses (authors Emma Mårdhed and Mats Zaar) with CBT and RP training, and psychiatric examinations are carried out by the first author (Anders Håkansson) of this paper. To a certain degree, psychiatric treatment other than gambling treatment is provided as part of the therapeutic work, or through medical treatment or referrals, whereas certain specific disorders (attention-deficit/hyperactivity disorder, psychotic disorders, or disorders with a comparable severity) may be treated in parallel with specialized psychiatric facilities.

Results were reported as descriptive data. Statistical associations were calculated between gambling types, presence of a psychiatric disorder, gender, and age, and these calculations were made using chi-square test for categorical data and Mann–Whitney *U* test for continuous variables. Statistical calculations were carried out in SPSS, version 22.0. In the analysis of associations with gambling types, subjects were excluded if this information was missing in the chart records.

The study was approved by the regional ethics committee, Lund, Sweden (file number 2017/341).

## Results

During the study period, 106 individuals contacted the facility in order to apply for treatment for problem gambling, and showed up for formal assessment. Seventy-nine percent (*n* = 84) sought treatment themselves, whereas 14% (*n* = 15) were referred from other psychiatric facilities, and 7% (*n* = 7) from primary care.

The median age of included patients was 31.5 years (range 18–72), and 80% of the patients (*n* = 85) were men. The Mann–Whitney *U* test demonstrated a nearly significant difference in age between women (median = 43 years, *n* = 21) and men (median = 30 years, *n* = 85), *p* = 0.05. Problematic gambling types were available from the records in 95 cases, and a clearly dominating gambling type was available from the records in 91 cases. The distribution of problem gambling types is displayed in Table 1. Among problem gambling types reported, the dominating gambling type was online casino in 55% of cases (*n* = 50), online sports betting in 24% (*n* = 22), land-based EGMs in 4% (*n* = 4), land-based casino in 3% (*n* = 3), and other games (such as online poker or bingo) in the remaining 13% (*n* = 12).

**Table 1:** Problem games reported from treatment-seeking patients (data available from 95 patients), not mutually exclusive.

Problem games reported	% (*n*)
Online casino	60% (*n* = 57)
Online sports betting	41% (*n* = 39)
Online casino or online sports betting	84% (n = 80)
Land-based casino	19% (*n* = 18)
Horse racing and sports games	8% (*n* = 8)
Electronic gambling machines	7% (*n* = 7)
Online poker	11% (*n* = 10)
Other	9% (*n* = 9)
*Any online gambling*	89% (*n* = 85)

Total	100% (*N* = 95)

Fifty-eight percent (*n* = 61) received a psychiatric diagnosis other than pathological gambling (all F diagnoses), and these disorders are displayed in Table 2. Non-substance-related psychiatric diagnoses (F diagnoses other than F10–F19) were present in 52% (*n* = 55). The chi-square test demonstrated that the difference in psychiatric comorbidity between genders only approached statistical significance (χ^2^ = 3.73, df = 1, *p* = 0.054); a comorbid diagnosis was present in 76% of females and 53% of males. For non-substance-related psychiatric comorbidity, the chi-square test demonstrated a significant association between gender and comorbidity (χ^2^ = 6.20, df = 1, *p* = 0.01), as this comorbidity was more common in women (76%) than in men (46%).

**Table 2:** Co-morbid psychiatric disorders (F disorders, in addition to pathological gambling, F63.0), according to ICD-10, not mutually exclusive.

Psychiatric disorder (other than pathological gambling, F63.0)	% (*n*)
No diagnosis	42% (*n* = 45)
Mental and behavioral disorders due to psychoactive substance use	12% (*n* = 13)
– alcohol (F10–)	7% (*n* = 7)
– drugs (F11–F19)	8% (*n* = 9)
– opioids (F11–)	3% (*n* = 3)
– cannabis (F12–)	4% (*n* = 4)
– sedatives (F13–)	1% (*n* = 1)
– cocaine (F14–)	2% (*n* = 2)
– polydrug (F19–)	1% (*n* = 1)
Mood disorders (F3–)	22% (*n* = 23)
– bipolar affective disorder (F31–)	3% (*n* = 3)
– depressive disorder (F32–)	20% (*n* = 21)
Neurotic, stress-related and somatoform disorders (F4–)	29% (*n* = 31)
– other anxiety disorders (F41–)	18% (*n* = 19)
– obsessive-compulsive disorder (42–)	1% (*n* = 1)
– reactions to severe stress, and adjustment disorders (F43–)	14% (*n* = 15)
– somatoform disorders (F45–)	1% (*n* = 1)
Disorders of adult personality and behavior (F6–)	5% (*n* = 5)
Disorders of psychological development (F8–)	2% (*n* = 2)
Behavioral and emotional disorders with onset usually occurring in childhood and adolescence (ADHD, ADD, F9–)	12% (*n* = 13)
Any psychiatric disorder	58% (*n* = 61)
Any non-substance psychiatric disorder	52% (*n* = 55)

Total	100% (*N* = 106)

The chi-square test demonstrated a significant association between problem online casino gambling and gender (χ^2^ = 13.95, df = 1, *p* < 0.001). Problem online casino gambling was more common in females (95%) than in male patients (50%). Also, with the chi-square test, online sports betting was significantly associated with gender (χ^2^ = 18.78, df = 1, *p* < 0.001), as this was reported by 53% of males and by none of the females. No significant association was seen between psychiatric comorbidity and problem online casino gambling (χ^2^ = 2.23, df = 1, *p* = 0.13). A psychiatric disorder was present in 63% of online casino clients and in 47% in others.

## Discussion

This study described the treatment-seeking patient population from the first 16 months of the first health care-based unit for pathological gambling treatment in a Swedish region, in order to describe patient characteristics in a situation where the health care system opens its first treatment option for this disorder in the region. The main finding is the overwhelming majority of treatment-seeking patients who report online casino or online sports betting to be one of their problem games, or their predominating problem game. In addition, the study lends support to previous findings of a high degree of psychiatric comorbidity in pathological gambling patients and demonstrated potentially higher comorbidity in treatment-seeking women than in men.

The high rates of online gambling in this population stand out compared to several reports from other previous treatment settings. For example, in a large material of treatment-seeking gambling patients in Barcelona, Internet-based gambling, as well as sports betting specifically, was uncommon (21), and Internet-based games constituted only a few percent of problem gambling in helpline callers in a US study (22), 5% of helpline callers in South Africa (23), and 16–17% of helpline users in an Australian study (24). Thus, the high proportion of online gambling—especially the high rates of online casino as the sole game or one of the problematic games reported by patients—stands out in this cohort of treatment-seeking patients, as these rates reported above from other settings have been considerably lower but also as some data on treatment-seeking behavior have indicated that online gambling may in fact be associated with a lower likelihood of seeking treatment (5), i.e., in contrast to the figures seen here.

The high rates of online gambling in treatment-seeking gamblers may be seen as worrying; problem gambling online may be associated with the use of unregulated sites (7), potentially making problem prevention more difficult. Also, although not a consistent finding, several reports have documented a potentially higher degree of problem gambling in online gamblers (11–14). The rates of non-online games were very low in this study, indicating that the predominance of online gambling may be specific to this setting. A recent government evaluation and bill proposal investigated the gambling market of Sweden and reported a clearly increasing market share for gambling companies operating online and overseas, and outside Swedish oligopoly based regulations for gambling. This share of the gambling market was reported to have increased dramatically over the past few years, including vast exposure in media commercials (10). Thus, while the high rates of online gambling in this treatment-seeking cohort may be surprising in comparison to previous publications from other settings, they clearly display an interesting picture of real-world gambling patients showing up when gambling treatment is started, in a setting exposed to large amounts of online gambling commercials.

While online gambling was the overwhelmingly most common type of game in this study, its association with gender differed depending on the type of game. Previously, online gambling has been associated with male gender (12, 25). Here, the same association was seen for online sports betting, whereas Internet casino was clearly associated with female gender; in fact, male and female patients in this cohort represented clearly different patterns of online gambling, especially as almost all females reported online casino, whereas none of them reported online sports betting.

The study of psychiatric comorbidity in different modalities of gambling is a relatively recent research topic. Blaszczynski and coworkers reported a lower degree of psychological distress in gamblers engaging in online gambling (17). In contrast, in the previous study, rates of comorbidity were high in online gamblers; however, without showing a significant difference compared to other gamblers.

However, female gender was linked to higher rates of psychiatric comobidity. Treatment-seeking female gamblers previously have been reported to have a higher degree of psychiatric problems than their counterparts (20, 22, 26), and women may be more likely to gamble in response to psychological distress (27). Thus, this study further supports the need for psychiatric assessment in female problem gamblers. In this study, a majority of treatment-seeking patients were men, consistently with previous publications (28). An important finding from previous literature has been that although gambling is generally associated with male gender, progression from gambling onset to problem gambling may be more rapid in women (29), and that time from problem gambling to treatment was shorter in women (30). Also, gambling for the purpose of mood regulation has been reported to be more common in female online gamblers than among their male counterparts (31). These findings, along with the findings of higher comorbidity in women in this study and others, demonstrate the need for further research and focus on treatment of gambling in women.

### Limitations

This study has limitations; the study period was intentionally short, in order to describe the population reached when a new facility opens the doors of the health care system to the gambling area, but this limits the total number of patients, making further statistical analyses difficult. In addition, the study was retrospective and based on hospital records, and as a consequence of this naturalistic design, data on gambling types were lacking in some cases. For the same reason, data were not available on patients’ duration of problem gambling, or the extent of gambling, such as money spent. Also, in the section describing comorbid psychiatric disorders, historical diagnoses were not registered. Thus, an estimation of lifetime prevalence of comorbid disorders likely would have been higher than the prevalence reported here. This may be a limitation, although the authors intended to describe the clinical picture of patients in close association with the gambling treatment episode. Also, although the study is retrospective and refers to patients seeking treatment before the study was actually carried out, one cannot exclude a potential bias from the fact that the first author (Anders Håkansson) was also the physician responsible at the unit and therefore responsible for some part of diagnosing (although not for the part referring to diagnoses from other units).

## Conclusion

This study displays the characteristics of treatment-seeking individuals at a gambling treatment facility opening in a setting without prior health care-based treatment for problem gambling. The high rates of online gambling and psychiatric comorbidity shed light on the treatment needs of problem gamblers in a setting where treatment has been largely underdimensioned, and where gamblers are extensively exposed to online gambling commercials in media. Treatment of pathological gambling may need to address psychiatric comorbidity with a particular emphasis on female clients.

## Ethics Statement

The study was approved by the regional ethics committee, Lund, Sweden (file number 2017/341). Consistent with this permission, based on the study being a retrospective chart review, no consent procedure was carried out.

## Author Contributions

AH carried out assessment of the hospital records included, statistical analyses, and wrote the manuscript. EM ad MZ carried out assessments of treatment-seeking patients. All authors reviewed and approved the manuscript.

## Conflict of Interest Statement

The authors declare that the research was conducted in the absence of any commercial or financial relationships that could be construed as a potential conflict of interest.
